# Association of serum vitamin C levels with Asthma in adults: results of NHANES 2003–2006 and mendelian randomization study

**DOI:** 10.1186/s12890-023-02821-w

**Published:** 2024-01-02

**Authors:** Kang Wang, Lintao Zhao, Hu Luo, Caixia Deng, Liang Gong, Zhujun Chen

**Affiliations:** Department of Respiratory and Critical Care Medicine, The First Affiliated Hospital of the Army Medical University, No. 30 Gaotanyanzheng Road, Shapingba District, Chongqing, 400038 China

**Keywords:** Serum vitamin C, Adult Asthma, National health and nutrition examination survey, Mendelian randomization, Causality

## Abstract

**Background:**

The protective effect of vitamin C as an antioxidant against asthma in adults remains controversial. This study used an observational study and Mendelian randomization (MR) analysis to investigate the association between adult asthma and serum vitamin C levels.

**Methods:**

Using information from the National Health and Nutrition Examination Survey (NHANES) 2003–2006, we carried out an observational study. A multivariate logistic regression model was employed to examine the connection between adult asthma and serum vitamin C levels. We used the inverse-variance weighted (IVW) method of MR analysis as the primary method to analyze the causal effect of serum vitamin C levels on asthma in adults.

**Results:**

A total of 8,504 participants were included in the observational study, including 639 in the asthma group and 7,865 in the non-asthma group. Before sample weighting, serum vitamin C was associated with a reduced risk of asthma in adults (OR = 0.798, 95% CI: 0.673–0.945, *P* = 0.009). After sample weighting, serum vitamin C was not associated with adult asthma risk (OR = 0.829, 95% CI: 0.660 ~ 1.042, *P* = 0.104). MR analysis showed no causal relationship between serum vitamin C and adult asthma in either the UK Biobank (OR = 0.957, 95% CI: 0.871 ~ 1.053, *P* = 0.370) or FinnGen (OR = 0.973, 95% CI: 0.824 ~ 1.149, *P* = 0.750) cohorts.

**Conclusion:**

Our study did not support a causal association between serum vitamin C levels and adult asthma risk. The relationship between serum vitamin C and adult asthma requires further research.

**Supplementary Information:**

The online version contains supplementary material available at 10.1186/s12890-023-02821-w.

## Background

Asthma is a respiratory disorder of chronic nature, marked by persistent airway inflammation, reversible airflow constriction, and increased airway reactivity [1]. According to the results of the Global Burden of Disease Study 2019, 262.4 million people worldwide are diagnosed with asthma, ranking first among chronic respiratory diseases and bringing a heavy medical and economic burden to the world [2]. The occurrence of asthma in China’s population aged 20 and above stands at 4.2%, and the number is approximately 45.70 million [3]. Oxidative stress is characterized by the overproduction of oxygen-derived free radicals (oxidants) within cellular structures, which leads to disorders of intracellular redox balance [4]. Recent researches have indicated that oxidative stress is a significant factor in the onset and progression of asthma [5, 6]. It can lead to cellular oxidative damage and inflammatory responses, exacerbating airway inflammation and bronchial smooth muscle contraction.

As an antioxidant, vitamin C has the ability to neutralize oxygen free radicals, thus shielding cells against harm resulting from oxidative stress [7]. A randomized controlled trial (RCT) concluded that vitamin C supplementation provided a protective effect against exercise-induced airway narrowing in asthmatic subjects [6]. However, A meta-analysis of nine RCTs concluded that it was not sufficient to recommend a specific role for vitamin C in the treatment of asthma [8]. Therefore, exploring the correlation between vitamin C and asthma may provide a fresh angle on strategies for asthma prevention and management.

National Health and Nutrition Examination Survey (NHANES) is a nationally representative survey operated by the National Center for Health Statistics (NCHS), conducted every two years using stratified multistage probability sampling to select respondents in the United States. The advantage of this survey lies in the combination of questionnaire interviews, laboratory tests, and physical examinations [9]. Mendelian randomization (MR) study is based on the Genome-Wide Association Study (GWAS) to collect and process the summarized data needed for the study, and to select genetic variation tools from it [10]. Because genetic variations occur randomly during gamete formation, it is not susceptible to reverse causation and confounding factors. MR studies, which analyzed the correlation between exposure factors and outcomes from a genetic perspective, were relatively reliable and were considered to be a good alternative to RCT [10].

Hence, to assess the impact of serum vitamin C levels on the risk of adult asthma, in this study, we first conducted an observational study of a U.S. population from the NHANES database. Subsequently, the causal association between serum vitamin C levels and adult asthma was assessed using a two-sample MR analysis, aiming to establish a novel scientific foundation for the prevention and management of asthma.

## Methods

### Participants in NHANES

**Fig. 1:**
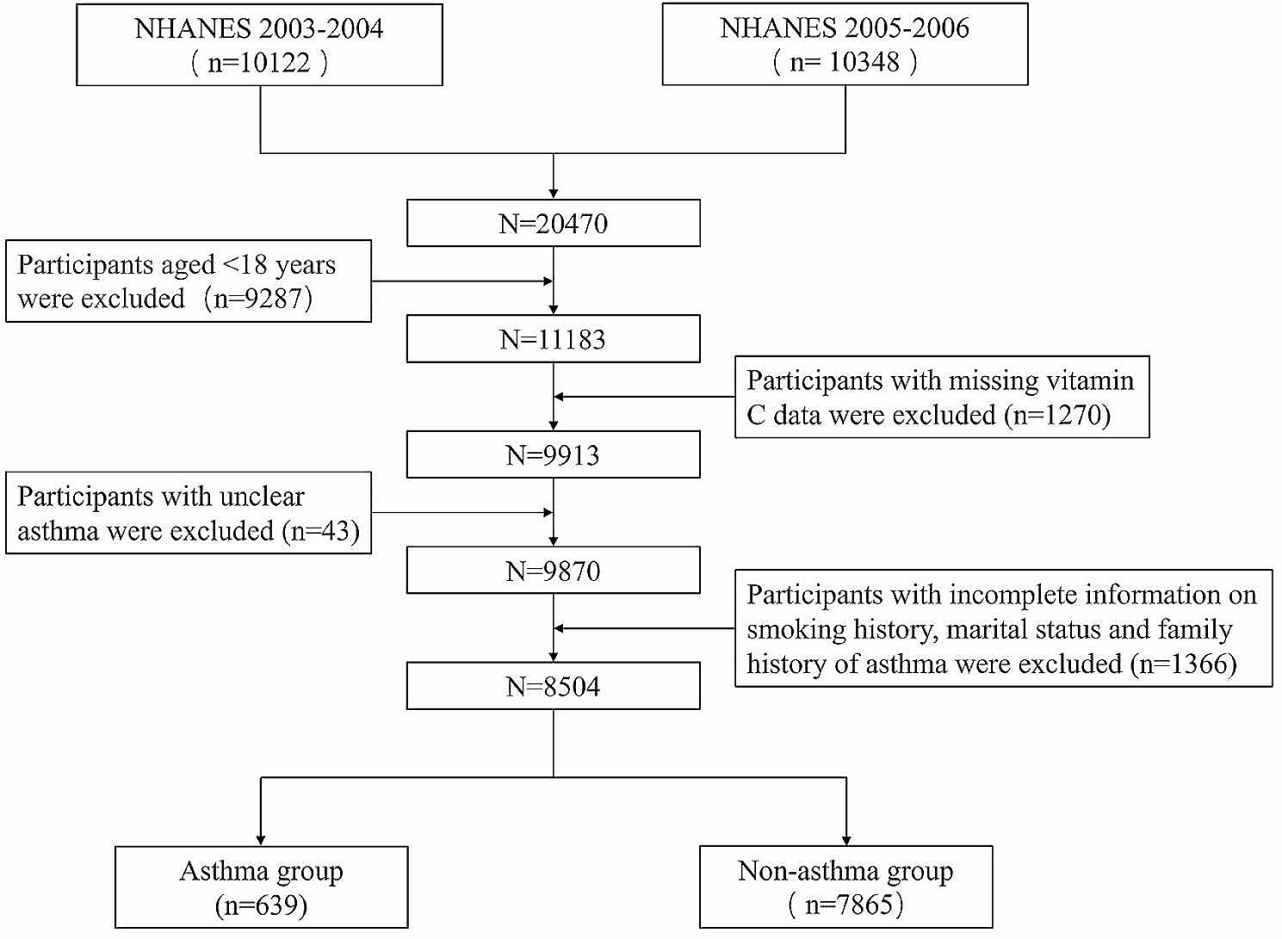
Flowchart of screening qualified participants for the observational study in NHANES 2003–2006. NHANES, National Health and Nutrition Examination Survey

NHANES 2003–2004 assessed 12,761 citizens, and 10,122 responded to the survey, with a response rate of 79.3%. NHANES 2005–2006 assessed 12,862 citizens, and 10,348 responded to the survey, with a response rate of 80.5%. We collected a total of 20,470 participants from the NHANES 2003–2004 and 2005–2006 (https://www.cdc.gov/nchs/nhanes/index.htm), and excluded participants who were younger than 18 years old, had lost serum vitamin C data, did not know whether they had asthma, and had incomplete clinical information. Finally, 8,504 participants were included in the study. Figure 1 illustrates the specific procedure.

### Definition of variables in NHANES

Asthma diagnosis relies on two specific questions from the Medical Condition questionnaire: (1) Ever been told you have asthma? (2) Still have asthma? If the participants answered YES for both questions, they were identified as asthma patients, otherwise, they were non-asthma patients. Family history of asthma is based on the question of “Blood relatives have asthma?“. Serum vitamin C levels were obtained from the Laboratory Data of NHANES and were measured using isocratic high-performance liquid chromatography (HPLC) with electrochemical detection at 650mV1. Demographic data (Gender, Age, Race, marital status) and lifestyle factors (smoking history) were obtained by questionnaire interview. Marital status was classified into two groups, married or living with partner (Married, Living with partner), unmarried or living alone (Widowed, Divorced, Separated, Never married). Smoking history was categorized as non-smoker (Smoked less than 100 cigarettes in life), former smoker (Smoked at least 100 cigarettes in life but does not smoke currently), and current smoker (Have Smoked at least 100 cigarettes in life and is still smoking).

### Genetic instrumental variables for serum vitamin C

**Fig. 2:**
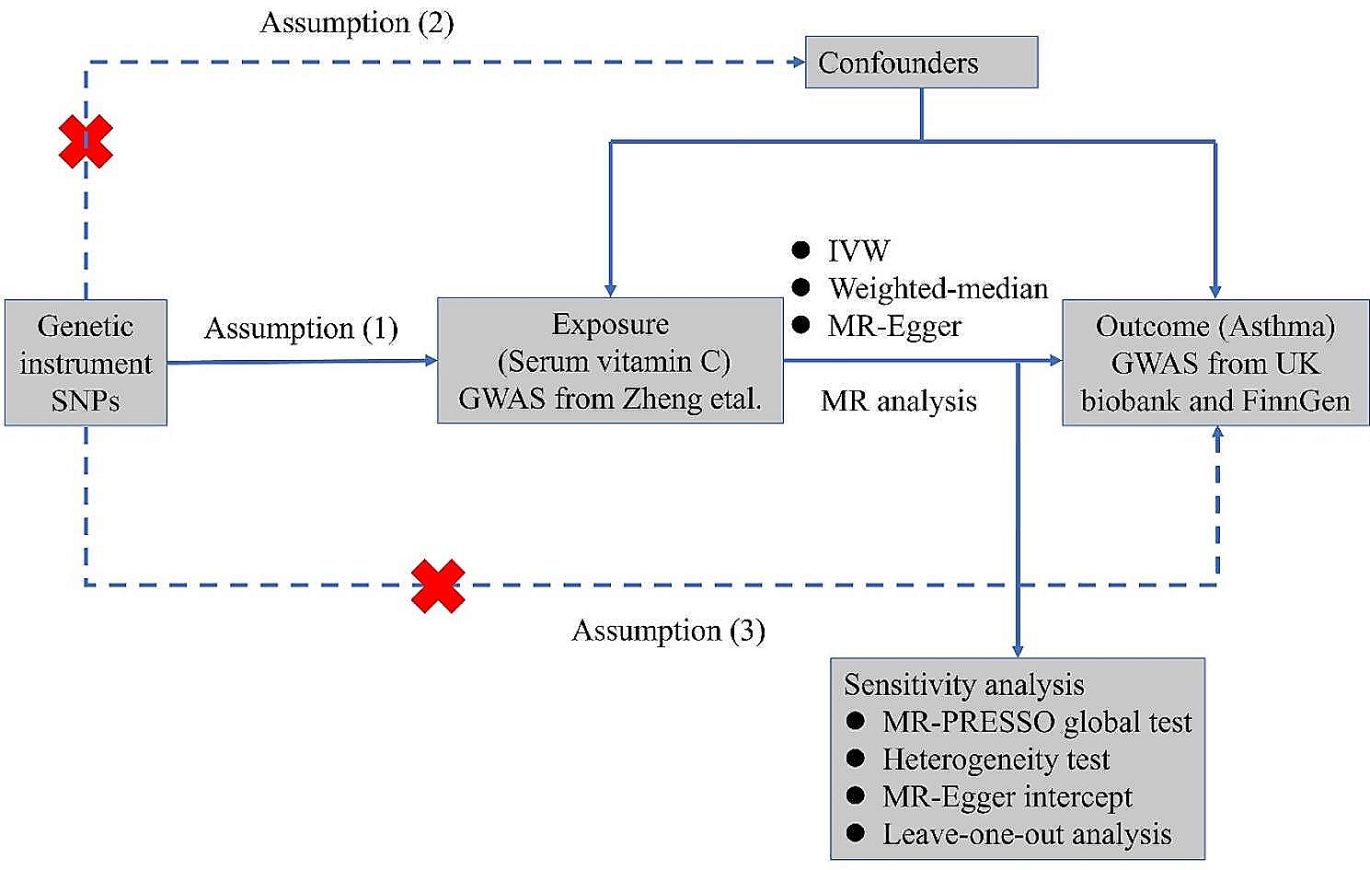
Mendelian randomization model and study overview. Red cross indicates that this pathway cannot be allowed. Assumption (1): There is a strong correlation between SNPs and exposure; Assumption (2): SNPs and confounders are independent of each other; Assumption (3): SNPs can only contribute to outcome through exposure. SNPs, single nucleotide polymorphisms; GWAS, genome wide association study; IVW, inverse-variance weighted; MR-PRESSO, Mendelian Randomization Pleiotropy RESidual Sum and Outlier

In this study, serum vitamin C was used as an exposure factor, and asthma as an outcome, and the causal relationship was analyzed by two-sample MR. Genetic instrumental variables (IVs) must satisfy three conditions: (1) IVs should be strongly correlated with serum vitamin C; (2) IVs cannot be directly associated with asthma and can only influence asthma through serum vitamin C; (3) IVs do not exhibit any associations with potential confounders. The F statistic is used to reflect the strength of each IV. To prevent bias resulting from weak IVs, F > 10 is required [11]. The formula for calculating the F-statistic for a single IV is as follows: F = R^2^(N-2)/(1-R^2^); R^2^=(2 × β^2^ × eaf×(1-eaf)) / [(2 × β^2^ × eaf×(1-eaf))+ (2×N×SE^2^×eaf×(1-eaf))]; Where N is the sample size in the exposure GWAS, the eaf is the effect allele frequency of the IVs, β is the beta value of the IVs, and SE is the standard error of β [12]. The specific process is shown in Fig. 2. We obtained genetic IVs from a GWAS meta-analysis of 52,018 European participants from Zheng et al. [13]. 11 single nucleotide polymorphisms (SNPs) related to serum vitamin C (Table S1) under the condition of genome-wide significance threshold *p* < 5 × 10^− 8^ were found in this study after adjusting for age, gender, research center, and other factors, these SNPs explained approximately 1.87% of the variation in serum vitamin C.

### GWAS data summary of Asthma

GWAS data on asthma are obtained from the UK biobank and FinnGen through the IEU open GWAS project (https://gwas.mrcieu.ac.uk). The UK biobank cohort (ID: ebi-a-GCST90014325) is a European population-based study in 2021 with 34,551,291 SNPs and 408,422 samples, of which 56,167 belonged to the case group, while 352,255 were part of the control group [14]. The FinnGen cohort (ID: finn-b-J10_ASTHMA) is also a European population-related study completed in 2021, with a total of 16,380,176 SNPs and 156,078 samples, including 20,629 in the case group and 135,449 in the control group (www.finngen.fi/en/). We searched for related phenotypes of 11 SNPs by PhenoScanner and found that SNP rs174547 was associated with asthma and was therefore excluded. No palindromic SNPs after harmonization. The relationship between SNPs of serum vitamin C and asthma is shown in Table S1.

### Statistical analysis

We conducted statistical analysis using R software (version 4.3.1). Before sample weighting, for non-normally distributed continuous variables, we presented them as median and quartile [M (P25, P75)] and employed the Mann-Whitney U test for group comparisons. As for categorical variables, we represented them using frequency and percentages (%), and conducted group comparisons using the chi-square test. Multivariate logistic regression was employed to compute the odds ratio (OR) and 95% confidence intervals (CIs) regarding the risk of adult asthma associated with serum vitamin C.

NHANES makes the data collected nationally representative through complex sampling designs and the use of sample weights, so we included primary sampling units (PSUs), stratification, and sampling weights in our analysis. After sample weighting, continuous variables were presented as means along with 95% CIs, and group comparisons were conducted using weighted *t*-tests. Categorical variables were represented as frequencies and weighted percentages (%), and group comparisons were performed using the weighted chi-square test. We employed a weighted Logistic regression model to assess the relationship between serum vitamin C levels and adult asthma.

In this study, the inverse-variance weighted (IVW) random effects model, weighted median, and MR Egger were used for MR analysis. The IVW method was the most powerful method to detect causality in two-sample MR Analysis, so we used it as our primary outcome. MR Egger and weighted median methods were used to supplement the estimation of IVW to make the results more reliable [15]. The results of the analyses were expressed as ORs, representing the risk of asthma per sd increase in serum vitamin C.

Cochran’s Q test [16] was performed on all SNPs to evaluate heterogeneity, with a *p* value > 0.05 and *I*^*2*^ < 25% representing no heterogeneity. When heterogeneity was present, the random effects model of IVW was used, otherwise, the fixed effects model of IVW was used. The MR-Egger method was employed to assess horizontal pleiotropy in SNPs, and if the intercept was close to zero (*p* > 0.05), there was no potential horizontal pleiotropy in SNPs [17]. Furthermore, we employed the MR-PRESSO method to identify horizontal pleiotropy and outliers, and the Global test *p* value > 0.05 suggested that there is no horizontal pleiotropy [18]. Leave-one-out analysis was conducted to examine if an individual SNP significantly affected the overall impact of serum vitamin C on adult asthma. We also assessed results stability through examining symmetry in the funnel plot. This study was analyzed by “TwoSampleMR” and “MRPRESSO” packages in R software (version 4.3.1). *p* < 0.05 was considered statistically significant.

## Results

### Basic characteristics of participants

The observational study included a total of 8,504 participants, there were 639 subjects in the asthma group and 7,865 in the non-asthma group. Before sample weighting, the two groups’ gender, race, marital status, family history of asthma, and serum vitamin C levels all showed statistically significant differences (*p* < 0.05). After sample weighting, differences in gender, race, and family history of asthma between the two groups were statistically significant (*p* < 0.05), while serum vitamin C levels did not exhibit a statistical difference (*p* > 0.05). See Table 1.

**Table 1:** Basic clinical characteristics of participants before and after sample weighting

Variables	Unweighted sample			Weighted sample		
	Asthma group (n = 639)	Non-asthma group (n = 7,865)	*p*	Asthma group (n = 639)	Non-asthma group (n = 7,865)	*p*
Gender			< 0.001			< 0.001
Male	234(36.6%)	3,862(49.1%)		234(36.1%)	3,862(49.1%)	
Female	405(63.4%)	4,003(50.9%)		405(63.9%)	4,003(50.9%)	
Age (years)	47 (32,63)	47 (32,65)	0.466	46.0 (44.2,47.9)	46.4 (45.5,47.4)	0.664
Race			< 0.001			< 0.001
Mexican American	72(11.3%)	1,673(21.3%)		72(4.0%)	1,673(8.4%)	
Other Hispanic	17(2.7%)	249(3.2%)		17(2.2%)	249(3.6%)	
Non-Hispanic White	370(57.9%)	4,018(51.1%)		370(77.1%)	4,018(71.9%)	
Non-Hispanic Black	155(24.3%)	1,608(20.4%)		155(11.8%)	1,608(11.0%)	
Other races	25(3.9%)	317(4.0%)		25(4.9%)	317(5.1%)	
Marital status			0.011			0.082
Married or living with a partner	370(57.9%)	4,952(63.0%)		370(61.1%)	4,952(66.2%)	
Unmarried or living alone	269(42.1%)	2,913(37.0%)		269(38.9%)	2,913(31.8%)	
Family history			< 0.001			< 0.001
Yes	327(51.2%)	1,490(18.9%)		327(50.7%)	1,490(19.7%)	
No	312(48.8%)	6,375(81.1%)		312(49.3%)	6,375(80.3%)	
Smoking history			0.243			0.792
Non-smoker	310(48.5%)	4,071(51.8%)		310(50.1%)	4,071(50.5%)	
Used to smoke	183(28.6%)	2,049(26.0%)		183(26.2%)	2,049(25.0%)	
Currently smoking	146(22.9%)	1,745(22.2%)		146(23.7%)	1,745(24.5%)	
Serum vitamin C (mg/dL)	0.91 (0.55,1.22)	0.97 (0.63,1.24)	0.015	0.92 (0.85,0.98)	0.96 (0.93,0.99)	0.126

### Associations between serum vitamin C and the prevalence of asthma

Before sample weighting, multivariate logistic regression was conducted in which gender, race, marital status, family history of asthma, and serum vitamin C level were considered to derive model 1, and model 2 was obtained after adjusting for age and smoking history. Serum vitamin C played a protective effect on adult asthma in both models 1 and 2 (*p* = 0.009, 0.007). After weighting the sample, we used weighted multivariate logistic regression. The findings from model 1 and 2 both indicated the absence of a statistically significant association between serum vitamin C and adult asthma occurrence (*p* > 0.05). See Table 2.

**Table 2:** Multivariate logistic regression of the associations between serum vitamin C and adult asthma

Models	Unweighted sample		Weighted sample	
	OR (95%CI)	*p*	OR (95%CI)	*p*
Model 1	0.798 (0.673,0.945)	0.009	0.829 (0.660,1.042)	0.104
Model 2	0.785 (0.658,0.935)	0.007	0.820 (0.653,1.030)	0.085

Model 2, adjusting for age and smoking history. OR, odds ratio; CI, confidence interval.

### Causal effect of serum vitamin C on adult Asthma

**Fig. 3:**
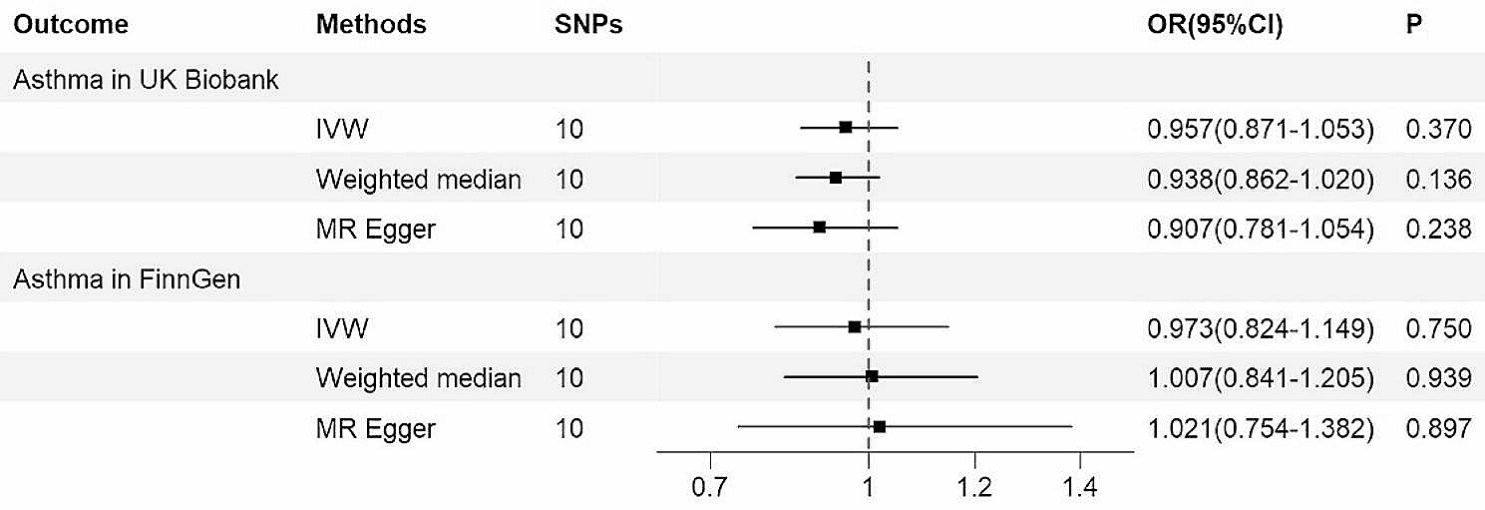
The MR findings regarding serum vitamin C levels and adult asthma. MR, mendelian randomization; SNPs, single nucleotide polymorphisms; IVW, inverse-variance weighted; OR, odds ratio

After excluding SNP rs174547, a total of 10 SNPs were analyzed for MR, and all of them had F-statistics > 10, thus avoiding significant bias due to the use of weak IVs. In the UK Biobank cohort, the IVW model showed that there was no causal relationship between serum vitamin C and adult asthma (OR = 0.957, 95% CI = 0.871–1.053, *p* = 0.370), and the MR-Egger and weighted median models also obtained similar results (Fig. 3). The MR analysis results of the FinnGen cohort were consistent with the above results (Fig. 3). Figure S1 shows the causality estimates for each of these 10 SNPs.

### Sensitivity analyses of MR

The *p* values of the MR-PRESSO global test for the UK Biobank and FinnGen cohorts were 0.081 and 0.127, respectively, indicating no horizontal pleiotropy and outlier for SNPs (Table S2). In the heterogeneity test, there was slight heterogeneity between SNPs in the UK Biobank and FinnGen cohorts (*p* = 0.031, 0.100; I^2^ = 50.9%, 38.7%). The intercepts of the MR-Egger regression for the UK Biobank and FinnGen cohorts were not significantly different from zero (intercept = 0.005, -0.004; *p* = 0.385, 0.716), indicating that there was no horizontal pleiotropy in SNPs (Fig. S2). In the leave-one-out analysis of Biobank and FinnGen, no SNPs strongly affected the overall effect of serum vitamin C on asthma (Fig. S3). Furthermore, the funnel plot was symmetrical, indicating the absence of pleiotropy (Fig. S4). The above sensitivity analysis results indicate that the MR analysis results are reliable, and the specific data are shown in Table S2.

## Discussion

We analyzed the association between serum vitamin C levels and adult asthma risk using a combination of observational study from NHANES 2003–2004 and 2005–2006, along with a two-sample MR analysis of GWAS data. According to our findings, no relationship exists between adult asthma and serum vitamin C levels.

Vitamin C serves as a potent antioxidant capable of neutralizing oxygen free radicals and mitigating oxidative damage. It is effective in the treatment and prevention of a variety of oxidative stress-related illnesses [19, 20]. However, whether vitamin C can reduce the risk of asthma remains controversial. Ben et al. [21] conducted a study on adult asthma, which involved 329 participants, consisting of 151 individuals in the asthma group and 178 in the healthy group. Their research examined systemic oxidant-antioxidant status in asthma patients and ultimately found that the asthma group had lower serum vitamin C levels. Shidfar et al. [22] conducted a case-control study in which they observed a significant decrease in serum vitamin C levels in the asthma group compared to the control group. In the asthma group, 38% of individuals had vitamin C deficiency (< 0.4 mg/dL), a significantly higher rate compared to the control group (0%). This suggests that low serum vitamin C levels may elevate the risk of asthma. Nevertheless, in a case-control study in adults by Picado et al. [23], serum vitamin C levels of 118 patients in the asthma group were (54 ± 17) µmol/L, and that of 121 patients in the control group was (58 ± 19) µmol/L, which were not statistically different, suggesting that serum vitamin C levels are not associated with asthma. Our results align with the research conducted by Picado et al. [23]. Observational studies not only have small sample sizes but also are easily interfered with by multiple confounding factors, leading to biased results. Hence, the findings of numerous observational investigations have been cast doubt upon. For instance, an observational study by Uysalol et al. [24] found that reduced vitamin D levels may elevate the susceptibility to childhood asthma, yet a meta-analysis of 32 studies by Beckhaus et al. [25] concluded that vitamin D is inconclusive for an effect on asthma. Meanwhile, a 2016 MR study concluded that diminished vitamin D levels are unlikely to cause asthma in children [26]. For this reason, we performed an exhaustive investigation regarding the association between serum vitamin C and the adult asthma risk based on the observational study from NHANES combined with MR analysis, and the results were more reliable.

NHANES is a US population-based survey that includes questionnaires, laboratory tests, and physical examinations [9]. Due to the use of stratified sampling, inconsistent data completeness of the participants, and the fact that only a small number of people participated in some specific programs, which can easily lead to biased results of the study, it is necessary to weigh the samples when conducting data analysis. At the same time, the weighted data are more truly reflective of the overall U.S. population [9]. Our study separately analyzed the impact of serum vitamin C on adult asthma before and after sample weighting. The results indicated that low serum vitamin C levels were an independent risk factor for the occurrence of adult asthma before sample weighting, but after sample weighting, there was no connection found between adult asthma and serum vitamin C levels. The results before and after sample weighting are completely opposite, so weighting the NHANES data is crucial. In the MR analysis, from the perspective of genetic susceptibility, we used 10 SNPs from GWAS data that are strongly related to serum vitamin C as IVs, and the random effects model of IVW was used as the primary outcome for assessing the causality of serum vitamin C and adult asthma. Finally, it was proved that there was no causal relationship between them, which was consistent with the results of the observational study from NHANES. Our results are consistent with a meta-analysis containing 9 RCTs by Kaur et al. [8]. Asthma is a multifaceted and intricate condition with a pathogenesis influenced by various factors, encompassing genetics, environment, immunity, and lifestyle [27]. Although vitamin C may influence the regulation of the immune system and oxidative stress, it is not a major determinant and therefore cannot explain the development of asthma alone. There may be a connection between vitamin C and pediatric asthma, according to several studies [28, 29]. The RCT of McEvoy et al. [28] noted that supplemental vitamin C taken by pregnant smokers improved newborn pulmonary function tests results and decreased wheezing through 1 year in the offspring. Nakamura et al. [29] investigated the relationship between vitamins and pediatric asthma, and their findings suggested that increased vitamin C intake might be associated with a decreased incidence of asthma. We believe that compared to adults, children are at a stage of growth and development where cell division and tissue repair are faster and require more vitamin C. Also, children usually have a higher metabolic rate and increased metabolic products (nitrogen radicals, reactive oxygen), so they need more antioxidants. For these reasons, vitamin C is linked to asthma in children but not adults.

The primary advantage of our research is that we combined the observational study with a two-sample MR to analyze the relationship between serum vitamin C levels and adult asthma risk. The observational study takes epidemiological factors into consideration, and the two-sample MR is explored from a genetic perspective, which can mitigate the influence of reverse causation and confounding factors, and the combination of the two can make the results more reliable. In addition, we used two independent cohorts from the UK Biobank and FinnGen databases for MR analysis, and their consistent results also increased the reliability of the MR Analysis. However, our research also comes with certain constraints. First, the observational study from NHANES is based on the American population, and the two-sample MR analysis is based on the European population. Differences in race may have some impact on the results, and future studies need to analyze the same ethnicity. Second, because the NHANES did not have data on serum vitamin C in recent years, we used data from 2003 to 2006, which may have affected the results. Finally, the genetic IVs obtained in European populations may not be present in other populations due to differences in genetic variants and allele frequencies across ethnicities, and thus our findings are only applicable to people of European descent. More observational and MR studies in other populations are needed in the future.

## Conclusions

In summary, our observational and MR studies did not support a causal relationship between serum vitamin C and adult asthma risk. Further exploration of the multifaceted origins of asthma is warranted in future research.

### Electronic supplementary material

Below is the link to the electronic supplementary material.

**Supplementary Material 1: Supplementary Table 1.** Genetic instruments for serum vitamin C and their associations with asthma from UK Biobank and FinnGen. **Supplementary Table 2.** Sensitivity analysis of mendelian randomization. **Supplementary Figure 1.** Forest plot of SNPs associated with serum vitamin C and their risk of adult asthma. **Supplementary Figure 2.** Scatter plot of SNPs associated with serum vitamin C and their risk of adult asthma. **Supplementary Figure 3.** Leave-one-out of SNPs associated with serum vitamin C and their risk of asthma. **Supplementary Figure 4.** Funnel plot of SNPs associated with serum vitamin C and their risk of asthma

**Supplementary Material 2:** STROBE-MR-checklist

## Data Availability

The data analyzed in this study are from the NHANES 2003–2006, which are publicly available and can be downloaded from the NHANES website (http://www.cdc.gov/nchs/nhanes.htm). The GWAS data of asthma were retrieved from IEU-Open GWAS project (https://gwas.mrcieu.ac.uk/datasets/ ebi-a-GCST90014325/ and https://gwas.mrcieu.ac.uk/datasets/ finn-b-J10_ASTHMA/).

## Abbreviations

MR: Mendelian randomization
NHANES: National health and nutrition examination survey
IVW: Inverse-variance weighted
OR: Odds ratio
CI: Confidence interval
RCT: Randomized controlled trial
NCHS: National center for health statistics
GWAS: Genome-Wide association study
HPLC: High-performance liquid chromatography
IVs: Instrumental variables
SNPs: Single nucleotide polymorphisms
MR-PRESSO: Mendelian randomization pleiotropy RESidual sum and outlier
PSUs: Primary sampling units
