# Supplementary material for: Association of serum vitamin C levels with Asthma in adults: results of NHANES 2003–2006 and mendelian randomization study
Source: BMC Pulm Med. 2024 Jan 2;24:4. doi: 10.1186/s12890-023-02821-w (PMC10759650; doi:10.1186/s12890-023-02821-w)

Supplementary Table 1: Genetic instruments for serum vitamin C and their associations with asthma from UK Biobank and FinnGen.

| SNPs | EA | OA | EAF | Exposure (Serum vitamin C) | | | | | | | | | | Outcome (Asthma in UK Biobank) | | | | | | | | Outcome (Asthma in FinnGen) | | | | | | | |
| --- | --- | --- | --- | --- | --- | --- | --- | --- | --- | --- | --- | --- | --- | --- | --- | --- | --- | --- | --- | --- | --- | --- | --- | --- | --- | --- | --- | --- | --- |
|  |  |  |  |  | N |  | Beta | SE | *p* | R^2^ |  | F |  |  | N |  | Beta | SE |  | *p* |  |  | N |  | Beta | SE |  | *p* |  |
| rs117885456 | A | G | 0.087 | 52018 | | | 0.078 | 0.012 | 1.70E-11 | 0.0008 | 42.25 | | | 408442 | | | 0.007 | 0.012 | 0.540 | | | 156078 | | | 0.017 | 0.021 | 0.420 | | |
| rs33972313 | C | T | 0.968 | 52018 | | | 0.360 | 0.018 | 4.61E-90 | 0.0076 | 399.98 | | | 408442 | | | -0.030 | 0.018 | 0.091 | | | 156078 | | | 0.026 | 0.040 | 0.527 | | |
| rs6693447 | T | G | 0.551 | 52018 | | | 0.039 | 0.006 | 6.25E-10 | 0.0008 | 42.25 | | | 408442 | | | -0.006 | 0.007 | 0.345 | | | 156078 | | | -0.011 | 0.012 | 0.326 | | |
| rs10051765 | C | T | 0.342 | 52018 | | | 0.039 | 0.007 | 3.64E-09 | 0.0006 | 31.04 | | | 408442 | | | 0.019 | 0.007 | 0.006 | | | 156078 | | | 0.024 | 0.012 | 0.041 | | |
| rs56738967 | C | G | 0.321 | 52018 | | | 0.041 | 0.007 | 7.62E-10 | 0.0007 | 34.30 | | | 408442 | | | 0.003 | 0.007 | 0.709 | | | 156078 | | | -0.004 | 0.013 | 0.746 | | |
| rs9895661 | T | C | 0.817 | 52018 | | | 0.063 | 0.008 | 1.05E-14 | 0.0012 | 62.01 | | | 408442 | | | -0.019 | 0.009 | 0.026 | | | 156078 | | | -0.017 | 0.015 | 0.255 | | |
| rs2559850 | A | G | 0.598 | 52018 | | | 0.058 | 0.006 | 6.30E-20 | 0.0018 | 93.44 | | | 408442 | | | 0.005 | 0.007 | 0.454 | | | 156078 | | | 0.011 | 0.012 | 0.347 | | |
| rs10136000 | A | G | 0.283 | 52018 | | | 0.040 | 0.007 | 1.33E-08 | 0.0006 | 32.65 | | | 408442 | | | -0.011 | 0.007 | 0.133 | | | 156078 | | | -0.024 | 0.013 | 0.063 | | |
| rs7740812 | G | A | 0.594 | 52018 | | | 0.038 | 0.006 | 1.88E-09 | 0.0008 | 40.11 | | | 408442 | | | 0.003 | 0.007 | 0.630 | | | 156078 | | | -0.002 | 0.013 | 0.896 | | |
| rs13028225 | T | C | 0.857 | 52018 | | | 0.102 | 0.009 | 2.38E-30 | 0.0025 | 128.44 | | | 408442 | | | -0.004 | 0.009 | 0.689 | | | 156078 | | | -0.024 | 0.012 | 0.041 | | |
| rs174547^a^ | C | T | 0.328 | 52018 | | | 0.036 | 0.007 | 3.84E-08 | 0.0005 | 26.45 | | | 408442 | | |  |  |  | | | 156078 | | |  |  |  | | |

^a^ rs174547 was associated with asthma and was therefore excluded. SNPs, single nucleotide polymorphisms; EA, effect allele; OA, other allele; EAF, effect allele frequency; SE, standard error.

Supplementary Table 2: Sensitivity analysis of mendelian randomization.

| Exposure | Outcome | MR-PRESSO | | |  | MR-Egger | | |  | Cochran Q test | | |  | Rucker’s Q’ test | |
| --- | --- | --- | --- | --- | --- | --- | --- | --- | --- | --- | --- | --- | --- | --- | --- |
|  |  | Casual estimate | sd | Global test *p* |  | intercept | se | *p* |  | Q value | *p* | I^2^ |  | Q value | *p* |
| Serum vitamin C | Asthma in UK Biobank | -0.043 | 0.048 | 0.081 |  | 0.005 | 0.006 | 0.385 |  | 18.346 | 0.031 | 50.9% |  | 16.592 | 0.035 |
| Serum vitamin C | Asthma in FinnGen | -0.027 | 0.085 | 0.127 |  | -0.004 | 0.010 | 0.716 |  | 14.674 | 0.100 | 38.7% |  | 14.417 | 0.072 |

MR-PRESSO, Mendelian Randomization Pleiotropy RESidual Sum and Outlier.

Supplementary Figure 1. Forest plot of SNPs associated with serum vitamin C and their risk of adult asthma. A: asthma from UK Biobank; B: asthma from FinnGen. MR, mendelian randomization; SNPs, single nucleotide polymorphisms.


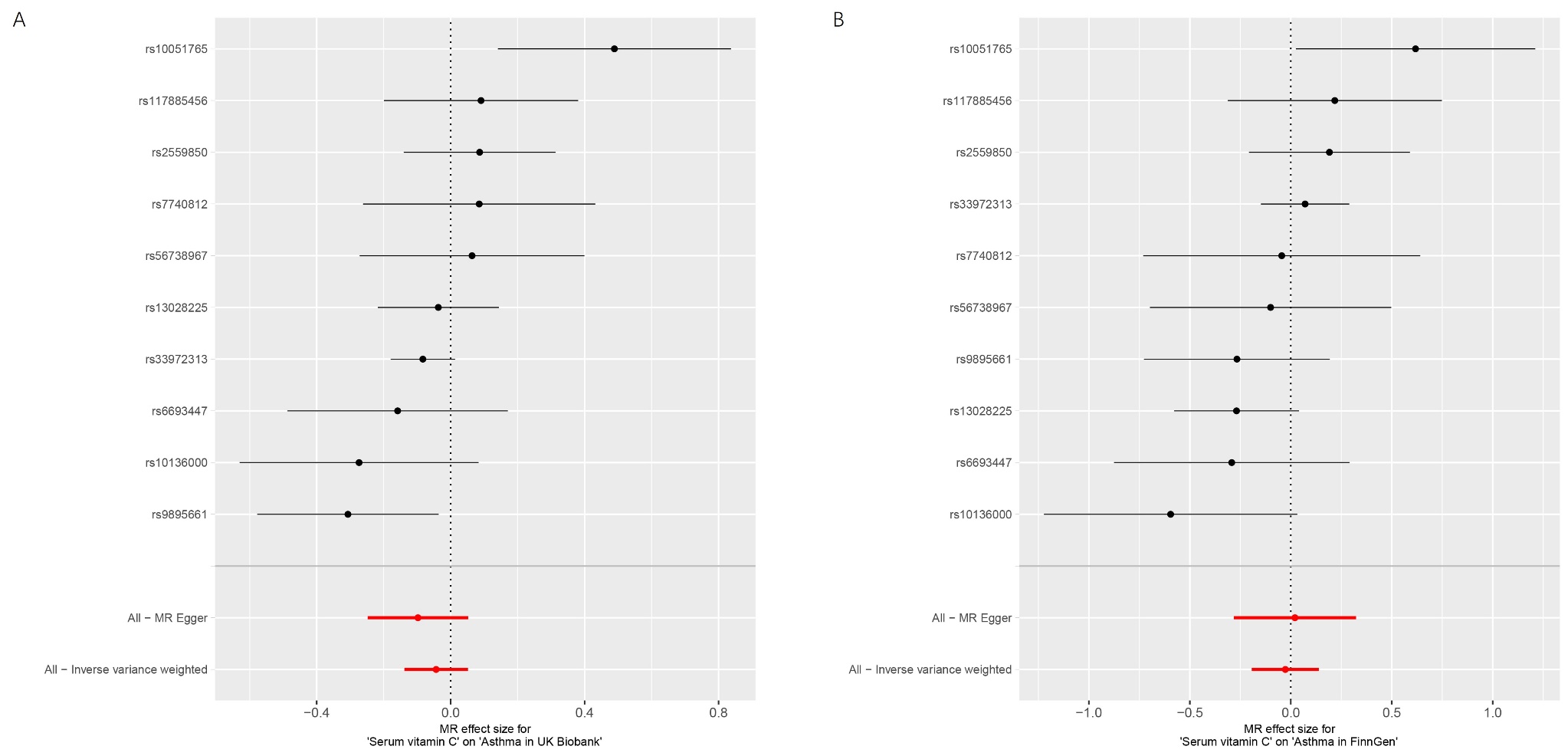


Supplementary Figure 2. Scatter plot of SNPs associated with serum vitamin C and their risk of adult asthma. A: asthma from UK Biobank; A: asthma from FinnGen. MR, mendelian randomization; SNPs, single nucleotide polymorphisms.


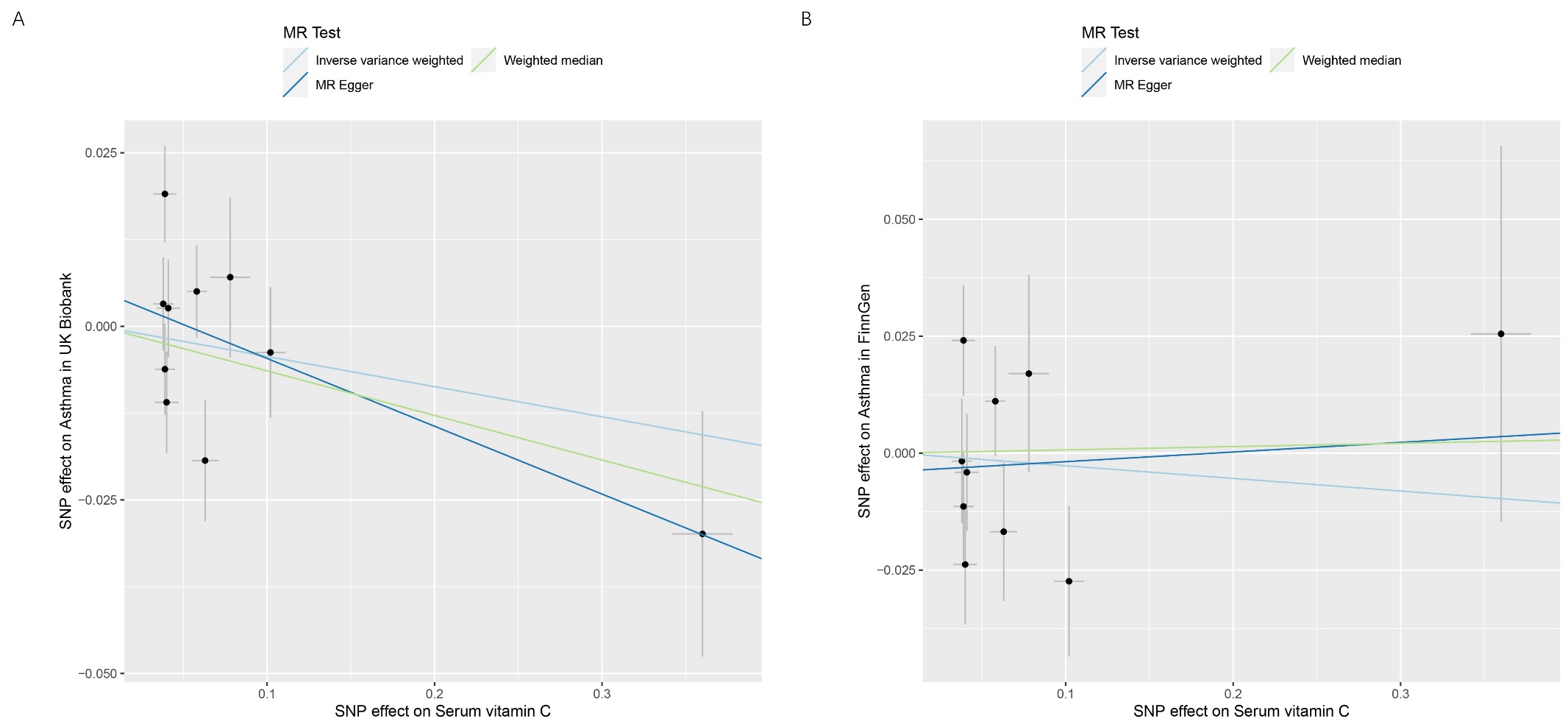


Supplementary Figure 3. Leave-one-out of SNPs associated with serum vitamin C and their risk of asthma. A: asthma from UK Biobank; A: asthma from FinnGen. MR, mendelian randomization; SNPs, single nucleotide polymorphisms.


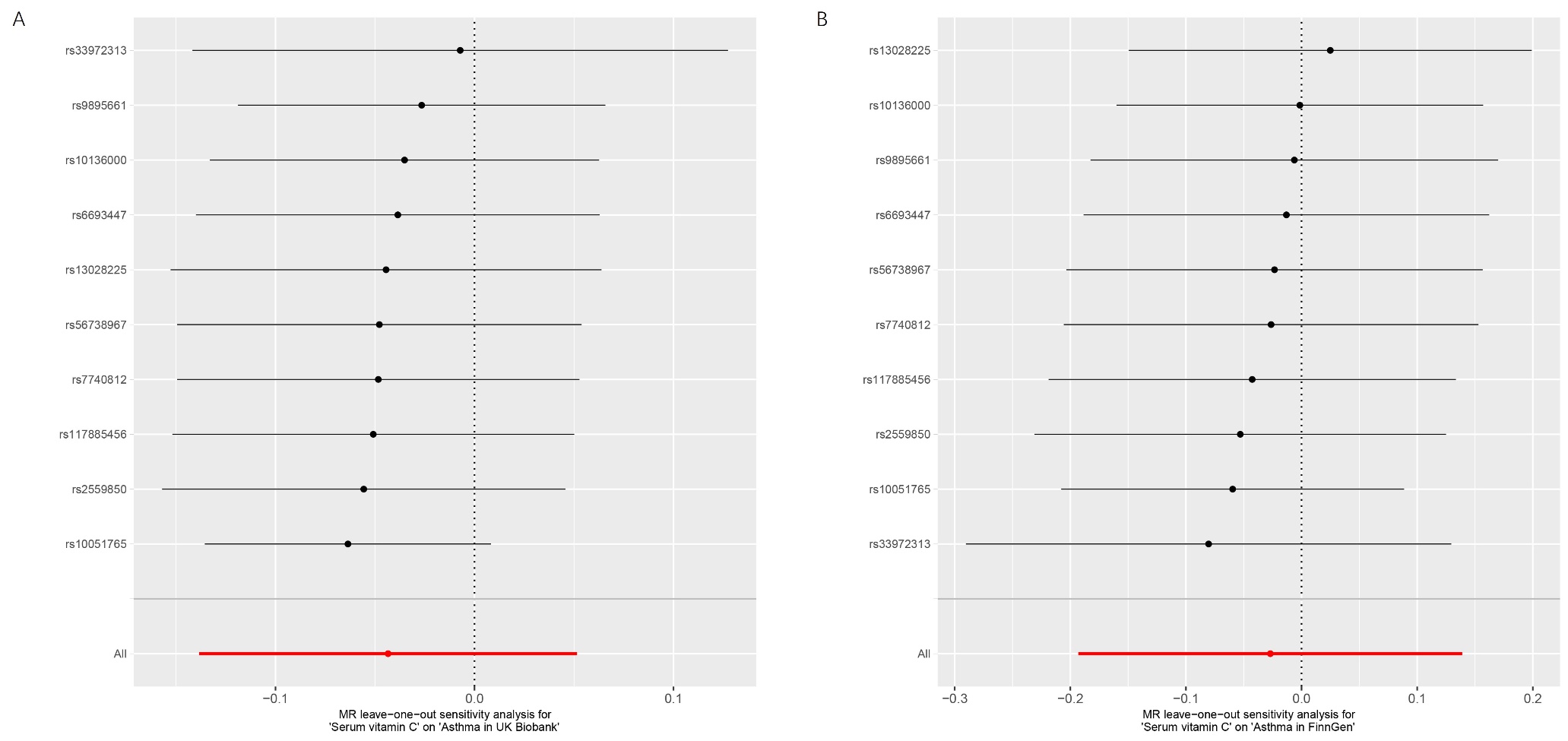


Supplementary Figure 4. Funnel plot of SNPs associated with serum vitamin C and their risk of asthma. A: asthma from UK Biobank; A: asthma from FinnGen. MR, mendelian randomization.


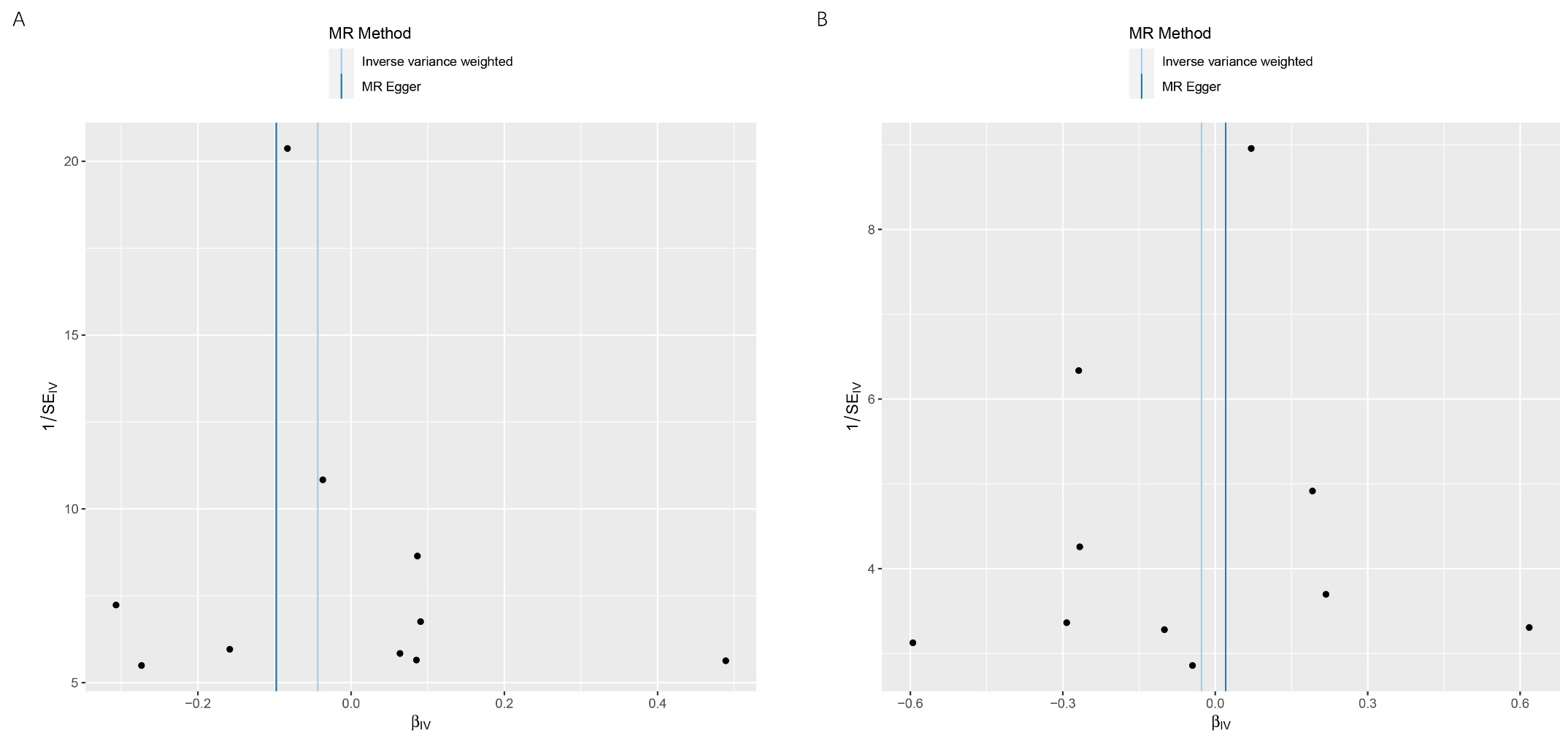

Supplement: Supplementary file 1 — Supplementary Material 1: Supplementary Table 1. Genetic instruments for serum vitamin C and their associations with asthma from UK Biobank and FinnGen. Supplementary Table 2. Sensitivity analysis of mendelian randomization. Supplementary Figure 1. Forest plot of SNPs associated with serum vitamin C and their risk of adult asthma. Supplementary Figure 2. Scatter plot of SNPs associated with serum vitamin C and their risk of adult asthma. Supplementary Figure 3. Leave-one-out of SNPs associated with serum vitamin C and their risk of asthma. Supplementary Figure 4. Funnel plot of SNPs associated with serum vitamin C and their risk of asthma [file 12890_2023_2821_MOESM1_ESM.docx]
